# 

**Published:** 1850-02

**Authors:** 


### WESTERN JOURNAL

OF

### MEDICINE AND SURGERY.

EDITORS.

---

LUNSFORD P. YANDELL, M. D.
PROFESSOR OF PHYSIOLOGY AND PATHOLOGICAL ANATOMY IN THE UNIVERSITY OF LOUISVILLE.

AND

THEODORE S BELL, M.D.

### THE MEDICAL JOURNAL FOR MARCH.

The numerous readers of this Journal will be gratified by the information we give them, that Prof. Bartlett is preparing an article for our March No. His paper is on a subject that is exceedingly interesting in itself; what it will be in his hands, it is scarcely necessary for us to say.

The Western Journal of Medicine and Surgery is published monthl[illegible] the undersigned, at the corner of Main and Fifth streets, Louisville, at $5 [illegible] annum, payable in advance. Each number contains 96 pages, making two [illegible]umes in the year of more than 550 pages each.

Contributions to its pages by the Physicians of the Valley of the Missis[illegible] addressed tothe Editors, are respectfully solicited.

Letters on business, to be addressed, postage paid, to the publishers.

Jauuary, 1844. PRENTICE & WEISSINGE[illegible]

### TRAVELING AGENTS FOR THE WESTERN JOURNAL OF MEDICINE AND SURGERY.

W. Ramsay, Kentucky—C. W. James Ohio, Indiana, Illinois, Mi[illegible] and Michigan—B. B. Brett, Tennessee—Wm. H. Weld & Son, Lou[illegible] and Arkansas—Morris & Co., Mississippi.

DELINQUENT SUBSCRIBERS.

This numerous class must see the necessity of making prompt remittances. We furnish a Journal which is not only a record of the progress of all the departments of Practical Medicine, but which contains the principles of practice adapted to the West and South. Its publication is attended with a heavy expenditure, and the subscribers should enable us to meet this expenditure without any trouble. We feel every assurance that we give them their money's worth, and we hope they will promptly respond to this call by remitting their dues.

PRENTICE & WEISSINGER, *Publishers.*

### *Receipts of Medical Journal for January,* 1850.

| | |
|---|---|
| Drs. Brockett & Hughes, Spencer, Tenn. | $3 00 |
| Yost & Frazier, Greenville, Ky. | 5 00 |
| Dr. H. Hoag, Lodi, Ohio, | 5 00 |
| W. Parker, New York, N. Y. | 10 00 |
| D. P. Stark, Haysville, Ky. | 5 00 |
| E. D. Forsee, Campbellsburg, Ky. | 5 00 |
| R. Burwell, Buffalo, N. Y. | 10 00 |
| J. Halstead, Lexington, Ky. | 5 00 |
| G. B. Miller, Fox, Iowa, | 5 00 |
| D. Thompson, McMillan's, Texas, | 5 00 |
| W. H. Lee, Columbia, Mo. | 5 00 |
| —— Murray, Laconia, Ark. | 10 00 |
| W. M. Dodson, Tuscumbia, Mo. | 5 00 |
| R. S. Wendell, Murfreesboro', Tenn. | 5 00 |
| C. W. Deane, Fayetteville, Ark. | 5 00 |
| W. Kenney, Millersburg, Ky. | 5 00 |
| J. J. Beatty, Gilbertsboro', Ala. | 30 00 |
| A. Gwathmey, Louisville, Ky. | 5 00 |
| W. C. Galt, " " | 5 00 |
| G. W. Bronough, Harrodsburg, Ky. | 5 00 |
| C. H. Spillman, Keene, Ky. | 5 00 |
| T. Lipscomb, Shelbyville, Tenn. | 5 00 |
| S. M. Clarkson, Halselville, S. C. | 5 00 |
| W. Brice, Youngsville, S. C. | 5 00 |
| C. C. E. Todd, Cap Au Grey, Mo. | 5 00 |
| N. A. Page, Conyersville, Tenn. | 5 00 |
| E. Harrison, Bryantsville, Ky. | 5 00 |

